# Beyond IMDC: do expanded models improve survival discrimination in metastatic renal cell carcinoma? A real-world cohort study

**DOI:** 10.3389/fonc.2026.1887870

**Published:** 2026-07-30

**Authors:** Erdoğan Şeyran, Azer Gökmen, Emre Hafızoğlu

**Affiliations:** 1 University of Health Sciences (SBI), Van Education and Research Hospital, Van, Türkiye; 2 Department of Medical Oncology, Republic of Türkiye (TC) Saglik Bakanligi Bursa Sehir Hastanesi, Nilüfer, Türkiye

**Keywords:** albumin, IMDC, metastatic renal cell carcinoma, neutrophil-to-lymphocyte ratio, prognostic modeling, survival discrimination

## Abstract

**Background:**

The International Metastatic Renal Cell Carcinoma Database Consortium (IMDC) classification remains the cornerstone of prognostic assessment in metastatic renal cell carcinoma (mRCC). However, whether metastatic burden and host-related inflammatory and nutritional biomarkers meaningfully improve prognostic discrimination beyond IMDC has not been fully established. This study aimed to evaluate whether expanded prognostic models incorporating metastatic burden, bone metastasis, neutrophil-to-lymphocyte ratio (NLR), albumin, and lactate dehydrogenase (LDH) improve survival discrimination beyond IMDC in a real-world mRCC cohort.

**Methods:**

We retrospectively evaluated 104 patients with mRCC treated at a single tertiary oncology center. Overall survival (OS), defined from the diagnosis of metastatic disease, was analyzed using Kaplan–Meier analysis and Cox proportional hazards regression models. Sequential multivariable Cox models were constructed by progressively adding metastatic burden, NLR, albumin, and LDH to the IMDC model. An alternative model incorporating bone metastasis status was also evaluated. Model performance was assessed using Harrell’s concordance index (C-index), −2 Log Likelihood statistics, and likelihood-ratio (LR) tests.

**Results:**

The cohort consisted predominantly of male patients (69.2%), with a median age of 60.3 years at metastatic diagnosis. According to IMDC classification, 24.0% of patients were favorable risk, 51.9% intermediate risk, and 24.0% poor risk. Median OS was 85.36 months in the favorable-risk group, 38.11 months in the intermediate-risk group, and 18.27 months in the poor-risk group (log-rank p<0.001). Elevated NLR was associated with significantly shorter OS in Kaplan–Meier analysis and with increased mortality in univariate Cox analysis (HR = 1.86, 95% CI 1.09–3.20, p=0.024), while higher serum albumin was associated with improved OS in univariate analysis (HR = 0.53, 95% CI 0.30–0.91, p=0.021). The fully expanded model demonstrated the highest discrimination (Harrell’s C-index 0.720), compared with 0.664 for the IMDC-only model. However, none of the sequential additions beyond IMDC significantly improved model performance according to likelihood-ratio testing, and metastatic burden, bone metastasis, NLR, albumin, and LDH did not retain independent prognostic significance after multivariable adjustment.

**Conclusions:**

In this real-world mRCC cohort, IMDC remained the dominant determinant of survival outcomes. Although expanded prognostic models incorporating metastatic burden and host-related biomarkers provided modest numerical improvements in model discrimination, they did not confer statistically significant incremental prognostic value beyond IMDC. These findings support the continued central role of IMDC for prognostic stratification in routine clinical practice.

## Introduction

Renal cell carcinoma (RCC) represents one of the most clinically significant urologic malignancies, accounting for approximately 2–3% of adult cancers worldwide (1). Despite advances in diagnosis and systemic treatment, a considerable proportion of RCC patients either present with metastatic disease at diagnosis or eventually develop distant metastases during follow-up (2). Systemic treatment strategies for metastatic RCC have undergone major changes over the past decade with the integration of immune checkpoint inhibitor (ICI)-based combinations and tyrosine kinase inhibitor (TKI)-based therapies, leading to improved survival outcomes across different risk groups (3, 4).

Reliable prognostic assessment remains critical for both survival estimation and treatment selection in patients with mRCC. Among currently available prognostic systems, the International Metastatic Renal Cell Carcinoma Database Consortium (IMDC) model remains the most extensively adopted prognostic system in routine clinical oncology practice (5). The IMDC model incorporates six clinical and laboratory parameters and stratifies patients into favorable, intermediate, and poor-risk categories (5). Importantly, IMDC classification continues to guide therapeutic decision-making in the era of ICI-based treatment algorithms and remains integrated into contemporary international guidelines (6, 7).

Despite the robust prognostic performance of IMDC, increasing evidence suggests that additional disease-related and host-related variables may further refine survival prediction in mRCC (8, 9). In recent years, systemic inflammatory and nutritional biomarkers have increasingly been investigated as potential adjunctive prognostic indicators because they may capture complex interactions between tumor-related inflammation, host immune response, and nutritional deterioration (10). Among these biomarkers, the neutrophil-to-lymphocyte ratio (NLR), serum albumin, and lactate dehydrogenase (LDH) have been associated with oncologic outcomes in several retrospective studies involving patients with mRCC (11–13).

In addition to laboratory-derived biomarkers, metastatic burden and metastatic pattern may also influence survival outcomes in mRCC (14). Previous studies have demonstrated that both the number and location of metastatic sites are associated with treatment response and overall survival, particularly in patients receiving modern systemic therapies (15). Bone metastasis has also been associated with more aggressive tumor biology, impaired quality of life, and inferior oncologic outcomes in several mRCC cohorts (16, 17).

However, although several studies have evaluated individual inflammatory, nutritional, and metastatic-pattern variables in mRCC, the extent to which these variables meaningfully enhance prognostic discrimination beyond IMDC remains uncertain in real-world clinical practice. Moreover, studies specifically assessing whether expanded prognostic models improve survival discrimination beyond IMDC using sequential model-based approaches remain limited.

Therefore, in the present real-world cohort study, we aimed to evaluate whether expanded prognostic models incorporating metastatic burden, bone metastasis, NLR, albumin, and LDH could improve survival discrimination beyond IMDC risk classification in patients with metastatic RCC. To address this question, we performed sequential Cox regression analyses and assessed incremental model performance using Harrell’s concordance index (C-index), likelihood-ratio (LR) tests, and model fit statistics.

## Materials and methods

### Study design and patient population

This retrospective cohort study included patients with metastatic renal cell carcinoma (mRCC) who were treated at the Department of Medical Oncology, Van Training and Research Hospital, Van, Türkiye, between January 2015 and January 2025. Patients who were initially diagnosed with renal cell carcinoma and subsequently developed metastatic disease, as well as those who presented with *de novo* metastatic disease, were screened for eligibility.

**Figure 1:**
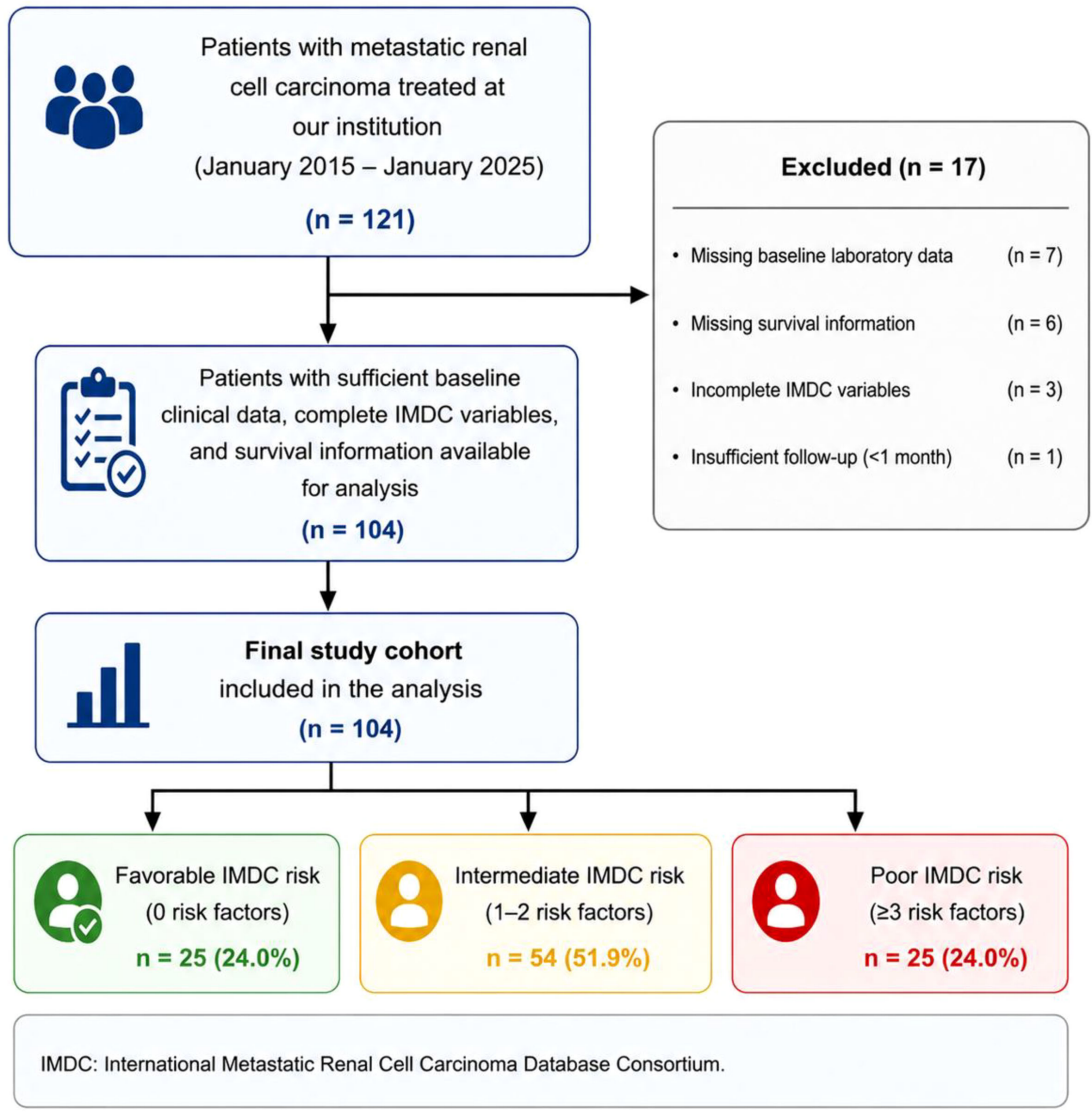
Flow diagram illustrating patient selection and cohort assembly for the study. IMDC risk categories are shown for the final study population.

A total of 121 consecutive patients were initially assessed. Patients were eligible if they were aged ≥18 years, had histopathologically confirmed renal cell carcinoma with metastatic disease, and had sufficient baseline clinicopathological and laboratory data available before initiation of first-line systemic therapy. Patients were excluded because of missing baseline laboratory data, missing survival information, incomplete IMDC variables, or insufficient follow-up data. After applying these eligibility criteria, 104 patients were included in the final analysis (**Figure 1**).

The study cohort comprised patients treated according to contemporary standards of care during the study period, including vascular endothelial growth factor receptor tyrosine kinase inhibitors (VEGFR-TKIs) and immune checkpoint inhibitor (ICI)-based therapies.

### Data collection

Demographic, clinical, pathological, and laboratory data were obtained through retrospective review of institutional electronic medical databases. Collected variables included age, sex, histological subtype, Eastern Cooperative Oncology Group (ECOG) performance status, IMDC risk classification, metastatic burden, metastatic sites, and survival status.

Metastatic burden was categorized as single-site or multiple-site metastatic disease. Metastatic sites included bone, liver, lung, and lymph node involvement. Histological subtype was classified as clear-cell or non-clear-cell RCC. First-line systemic treatment was categorized as tyrosine kinase inhibitor (TKI) monotherapy, immune checkpoint inhibitor plus TKI (ICI–TKI) combination therapy, or dual immune checkpoint inhibitor (ICI–ICI) combination therapy. Baseline laboratory parameters recorded prior to initiation of systemic therapy included white blood cell count (WBC), neutrophil count, lymphocyte count, platelet count, hemoglobin, serum albumin, lactate dehydrogenase (LDH), and neutrophil-to-lymphocyte ratio (NLR). Because no universally accepted cut-off values have been established for NLR, albumin, or LDH in metastatic renal cell carcinoma, and previous studies have reported heterogeneous threshold values depending on study design and patient population, cohort-specific median values were selected for dichotomization in this exploratory analysis. This approach is commonly used in exploratory retrospective biomarker studies when externally validated thresholds are unavailable. Accordingly, median NLR (3.15), albumin (3.85 g/dL), and LDH (227 U/L) values were used as cut-off points for dichotomized survival analyses.

### Outcome definition

Overall survival (OS) was the primary study endpoint and was defined as the interval from the date of metastatic disease diagnosis to death from any cause or last follow-up. The date of metastatic disease diagnosis was used as the time origin for all survival analyses. Patients who were alive at the last follow-up were censored.

### Statistical analysis

All statistical analyses were performed using IBM SPSS Statistics version 26.0 (IBM Corp., Armonk, NY, USA). Continuous variables are presented as median (range), whereas categorical variables are presented as frequencies and percentages.

Median follow-up was estimated using the reverse Kaplan–Meier method. Overall survival (OS) was analyzed using the Kaplan–Meier method, and survival differences between groups were compared using the log-rank test. Kaplan–Meier analyses were performed according to IMDC risk classification, metastatic burden, bone metastasis status, neutrophil-to-lymphocyte ratio (NLR), and serum albumin groups.

Univariate Cox proportional hazards regression analyses were performed to evaluate the association between clinicopathological variables and OS. Hazard ratios (HRs) and 95% confidence intervals (95% CIs) were reported. Variables included in the multivariable models were selected based on clinical relevance and prior literature.

To investigate the incremental prognostic value of expanded variables beyond IMDC, sequential multivariable Cox regression models were constructed by sequentially adding prespecified variables. Model 1 included only IMDC risk classification. Model 2 additionally incorporated metastatic burden. Model 3 included IMDC risk classification, metastatic burden, and NLR. Albumin was added in Model 4, while LDH was additionally incorporated in Model 5. An alternative model was constructed by replacing metastatic burden with bone metastasis status. Sequential multivariable analyses were performed using a common complete-case cohort to ensure direct comparability across all models.

Model performance was assessed using Harrell’s concordance index (C-index), −2 Log Likelihood statistics, and likelihood-ratio (LR) tests. Higher Harrell’s C-index values indicated better discriminatory performance, whereas lower −2 Log Likelihood values indicated better model fit. Approximate 95% confidence intervals (95% CIs) for Harrell’s C-index were estimated using 1,000 bootstrap resamples. Statistical significance was defined as a two-sided *p* value <0.05.

### Ethical approval

The study was approved by the Institutional Ethics Committee of Van Training and Research Hospital (Decision No: GOKAEK/2026-05/18, Date: 08.05.2026). The approval covered the retrospective analysis of previously collected patient data between January 2015 and January 2025. The study was conducted in accordance with the Declaration of Helsinki. Due to the retrospective design and use of anonymized data, informed consent was waived.

## Results

### Patient characteristics

The final study cohort consisted of 104 patients with metastatic renal cell carcinoma (mRCC). The median age at metastatic disease diagnosis was 60.3 years (range, 28.4–83.5 years), and 69.2% of the patients were male. Clear-cell histology was present in 86.5% of patients, whereas 13.5% had non-clear-cell RCC. According to the IMDC risk classification, 24.0% of patients were categorized as favorable risk, 51.9% as intermediate risk, and 24.0% as poor risk. Multiple-site metastatic disease was observed in 63.5% of patients. Lung metastasis was the most common metastatic site (70.2%), followed by bone (59.6%), lymph node (50.0%), and liver metastases (18.3%). Regarding first-line systemic treatment, 37 patients (35.6%) received TKI monotherapy, 45 (43.3%) received an ICI–TKI combination, and 22 (21.2%) received an ICI–ICI combination. The median follow-up, estimated using the reverse Kaplan–Meier method, was 64.1 months (95% CI, 44.0–90.2 months). Baseline demographic, clinicopathological, treatment, follow-up, and laboratory characteristics are summarized in **Table 1**.

**Table 1:** Baseline demographic, clinicopathological, treatment, follow-up, and laboratory characteristics of patients with metastatic renal cell carcinoma. .

Variable	Overall (n=104)
Age at metastatic diagnosis, years	60.3 (28.4–83.5)
Sex	
Male	72 (69.2)
Female	32 (30.8)
Histology	
Clear-cell RCC	90 (86.5)
Non-clear-cell RCC	14 (13.5)
IMDC risk classification	
Favorable	25 (24.0)
Intermediate	54 (51.9)
Poor	25 (24.0)
Metastatic burden	
Single-site	38 (36.5)
Multiple-site	66 (63.5)
Metastatic sites	
Bone	62 (59.6)
Liver	19 (18.3)
Lung	73 (70.2)
Lymph node	52 (50.0)
ECOG performance status	
0	4 (3.8)
1	61 (58.7)
2	30 (28.8)
3	9 (8.7)
First-line systemic therapy	
TKI monotherapy	37 (35.6)
ICI–TKI combination therapy	45 (43.3)
ICI–ICI combination therapy	22 (21.2)
Laboratory variables	
Hemoglobin, g/dL	12.2 (7.4–17.2)
Albumin, g/dL	3.85 (2.3–4.7)
LDH, U/L	227 (106–925)
NLR	3.15 (0.69–42.56)
Follow-up	
Median follow-up, months (95% CI)	64.1 (44.0–90.2)
Vital status	
Alive	48 (46.2)
Dead	56 (53.8)

RCC, renal cell carcinoma; IMDC, International Metastatic Renal Cell Carcinoma Database Consortium; ECOG, Eastern Cooperative Oncology Group; TKI, tyrosine kinase inhibitor; ICI, immune checkpoint inhibitor; LDH, lactate dehydrogenase; NLR, neutrophil-to-lymphocyte ratio.

Continuous variables are presented as median (range) and categorical variables as number (%). Age was calculated at the diagnosis of metastatic disease. Median follow-up was estimated using the reverse Kaplan–Meier method, with metastatic disease diagnosis used as the time origin. Laboratory data were available for 103 patients for hemoglobin, 103 for albumin, 104 for LDH, and 102 for NLR. Overall survival was defined as the interval from metastatic disease diagnosis to death from any cause or last follow-up. Patients may have had more than one metastatic site at diagnosis; therefore, percentages do not sum to 100%.

### Kaplan–Meier survival analyses

**Figure 2:**
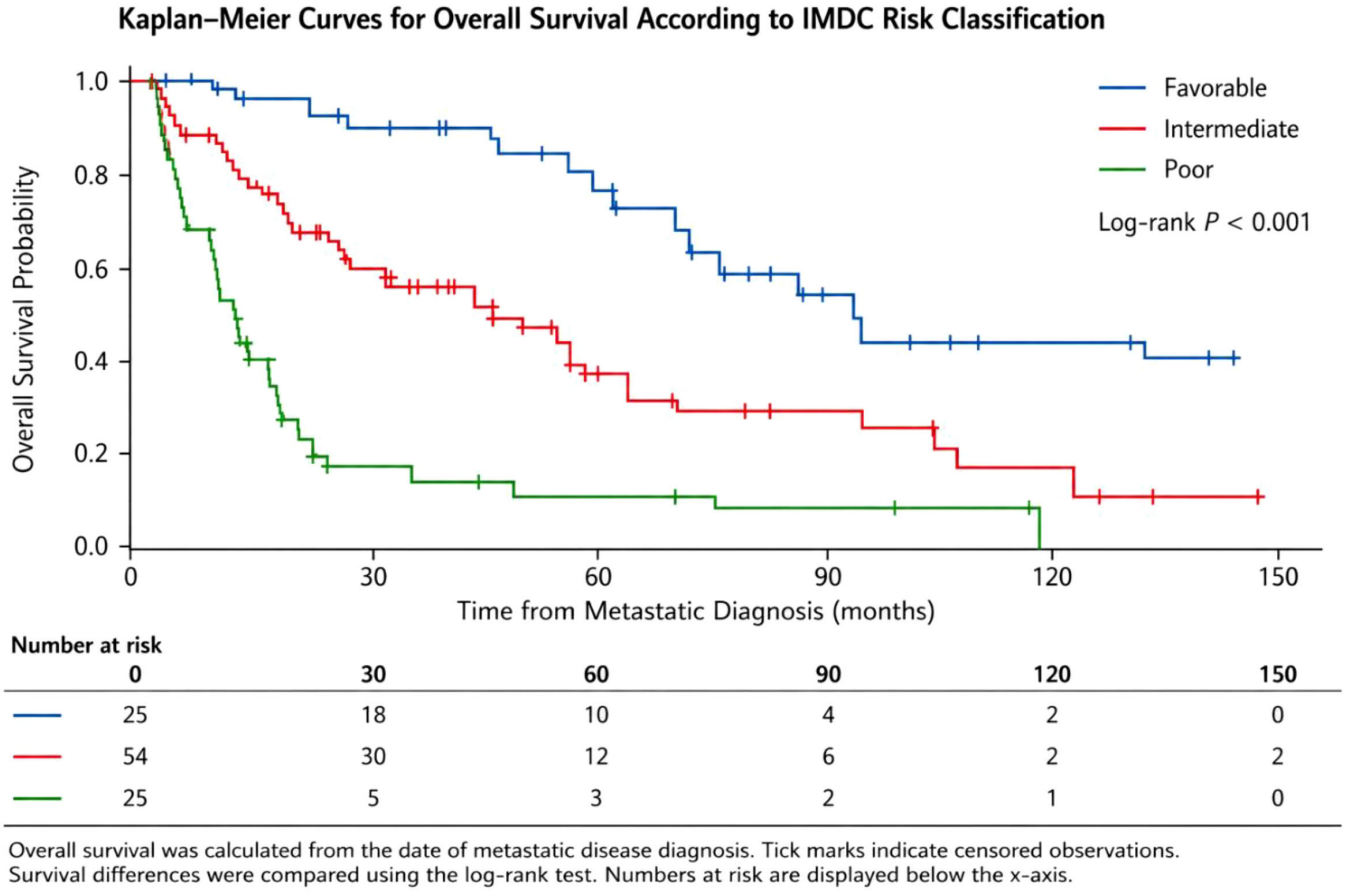
Kaplan–Meier curves of overall survival according to IMDC risk classification. Overall survival was calculated from the date of metastatic disease diagnosis. Tick marks indicate censored observations. P values were calculated using the log-rank test. Numbers at risk are displayed below the x-axis.

Kaplan–Meier survival analysis demonstrated significant differences in overall survival (OS) among IMDC risk groups (log-rank p<0.001). Median OS was 85.36 months in the favorable-risk group, 38.11 months in the intermediate-risk group, and 18.27 months in the poor-risk group (**Table 2**, **Figure 2**).

**Table 2:** Kaplan–Meier analysis of overall survival according to clinicopathological and laboratory variables.

Variable	n	Median OS (months)	Log-rank p-value
IMDC risk classification	104		**<0.001**
• Favorable	25	**85.36**	
• Intermediate	54	**38.11**	
• Poor	25	**18.27**	
Metastatic burden	104		**0.054**
• Single-site metastasis	38	**65.02**	
• Multiple-site metastasis	66	**34.20**	
Bone metastasis	104		**0.187**
• Absent	42	**61.90**	
• Present	62	**37.29**	
Neutrophil-to-lymphocyte ratio (NLR)	102		**0.022**
• Low (<3.15)	51	**85.36**	
• High (≥3.15)	51	**34.20**	
Albumin	103		**0.019**
• Low (<3.85 g/dL)	56	**30.49**	
• High (≥3.85 g/dL)	47	**78.82**	

OS, overall survival; IMDC, International Metastatic Renal Cell Carcinoma Database Consortium; NLR, neutrophil-to-lymphocyte ratio.

Overall survival was defined as the interval from the diagnosis of metastatic disease to death from any cause or last follow-up. Survival curves were estimated using the Kaplan–Meier method and compared using the log-rank test. NLR and albumin were dichotomized according to the prespecified analysis cut-offs (NLR 3.15 and albumin 3.85 g/dL). Bold values indicate median OS estimates and log-rank p-values.

**Figure 3:**
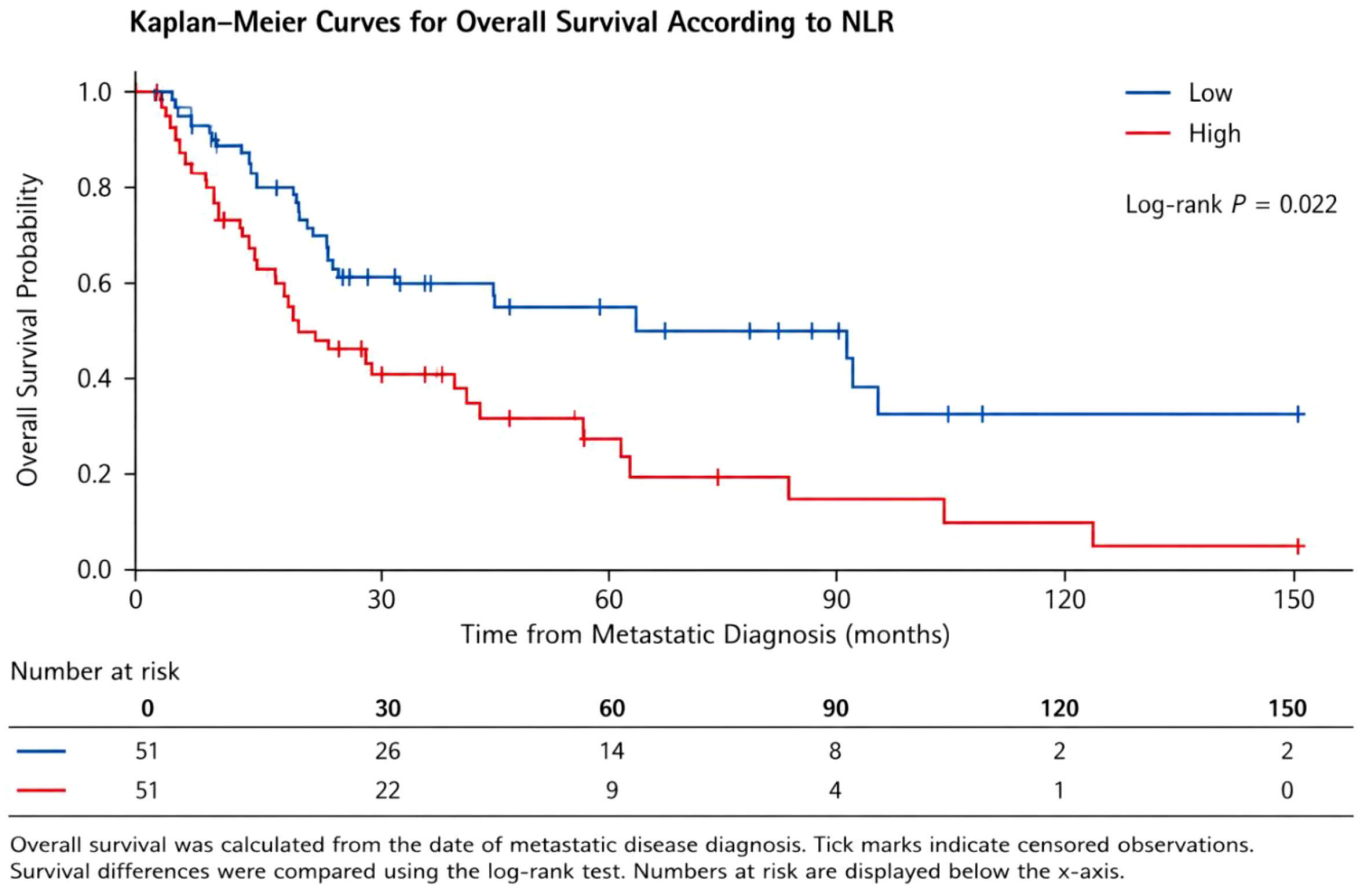
Kaplan–Meier curves of overall survival according to baseline neutrophil-to-lymphocyte ratio (NLR). Overall survival was calculated from the date of metastatic disease diagnosis. Tick marks indicate censored observations. P values were calculated using the log-rank test. Numbers at risk are displayed below the x-axis.

Patients with low NLR demonstrated significantly longer OS than those with high NLR (median OS, 85.36 vs. 34.20 months; log-rank p=0.022) (**Table 2**, **Figure 3**).

Patients with higher serum albumin levels also demonstrated significantly longer OS than those with lower albumin levels (median OS, 78.82 vs. 30.49 months; log-rank p=0.019) (**Table 2**).

No statistically significant survival differences were observed according to metastatic burden or bone metastasis status. Although patients with single-site metastatic disease and those without bone metastasis showed numerically longer OS, these differences did not reach statistical significance (**Table 2**).

### Univariate Cox regression analysis

In univariate Cox regression analysis, IMDC risk classification was significantly associated with overall survival. Compared with the favorable-risk group, patients in the poor-risk category had a 4.31-fold higher risk of mortality (HR = 4.31, 95% CI: 1.91–9.73, p<0.001), whereas the increased mortality risk observed in the intermediate-risk group did not reach statistical significance (HR = 1.85, 95% CI: 0.87–3.91, p=0.109) (**Table 3**).

**Table 3:** Univariate Cox regression analysis for overall survival.

Variable	Hazard ratio (95% CI)	P value
IMDC risk classification		
Intermediate vs. Favorable	1.85 (0.87–3.91)	0.109
Poor vs. Favorable	4.31 (1.91–9.73)	**<0.001**
Metastatic burden		
Multiple vs. Single	1.78 (0.98–3.24)	0.057
Bone metastasis		
Present vs. Absent	1.46 (0.83–2.57)	0.190
Neutrophil-to-lymphocyte ratio		
High (≥3.15) vs. Low (<3.15)	1.86 (1.09–3.20)	**0.024**
Albumin		
High (≥3.85 g/dL) vs. Low (<3.85 g/dL)	0.53 (0.30–0.91)	**0.021**
LDH		
High (≥227 U/L) vs. Low (<227 U/L)	1.44 (0.84–2.50)	0.188

HR, hazard ratio; CI, confidence interval; IMDC, International Metastatic Renal Cell Carcinoma Database Consortium; LDH, lactate dehydrogenase; NLR, neutrophil-to-lymphocyte ratio.

Reference categories were favorable IMDC risk, single-site metastasis, absence of bone metastasis, low NLR (<3.15), low albumin (<3.85 g/dL), and low LDH (<227 U/L). Overall survival was defined as the interval from the diagnosis of metastatic disease to death from any cause or last follow-up. Analyses involving NLR and albumin were performed on patients with available laboratory data (n=102 and n=103, respectively). Bold values indicate statistically significant results (p < 0.05).

Elevated NLR was significantly associated with worse overall survival in univariate analysis (HR = 1.86, 95% CI: 1.09–3.20, p=0.024). Conversely, higher serum albumin levels were significantly associated with improved overall survival (HR = 0.53, 95% CI: 0.30–0.91, p=0.021).

Multiple-site metastatic disease demonstrated a borderline association with poorer overall survival (HR = 1.78, 95% CI: 0.98–3.24, p=0.057), whereas bone metastasis (HR = 1.46, 95% CI: 0.83–2.57, p=0.190) and elevated LDH (HR = 1.44, 95% CI: 0.84–2.50, p=0.188) were not significantly associated with overall survival (**Table 3**).

### Sequential multivariable Cox regression models and model performance

Sequential multivariable Cox regression analyses demonstrated that IMDC risk classification remained the dominant independent prognostic factor across all models (**Table 4**). Compared with the favorable-risk group, poor IMDC risk remained independently associated with significantly worse overall survival throughout the sequential models, with hazard ratios ranging from 4.12 (95% CI: 1.77–9.62, p=0.001) in Model 1 to 2.77 (95% CI: 1.12–6.87, p=0.028) in Model 5. In contrast, intermediate IMDC risk was not independently associated with overall survival in any multivariable model (all p>0.05).

**Table 4:** Sequential multivariable Cox regression models for overall survival and model performance.

Variable	Model 1	Model 2	Model 3	Model 4	Model 5	Alternative model
	IMDC	IMDC + metastatic burden	Model 2 + NLR	Model 3 + albumin	Model 4 + LDH	IMDC + bone metastasis + NLR + albumin
IMDC intermediate vs. favorable	1.70 (0.77–3.76), *p* = 0.190	1.61 (0.73–3.57), *p* = 0.241	1.53 (0.69–3.41), *p* = 0.294	1.35 (0.59–3.11), *p* = 0.479	1.34 (0.58–3.09), *p* = 0.496	1.32 (0.59–2.91), *p* = 0.498
IMDC poor vs. favorable	4.12 (1.77–9.62), *p* = 0.001	3.72 (1.58–8.76), *p* = 0.003	3.15 (1.31–7.55), *p* = 0.010	2.82 (1.14–6.96), *p* = 0.025	2.77 (1.12–6.87), *p* = 0.028	2.86 (1.20–6.82), *p* = 0.018
Multiple-site vs. single-site metastasis	—	1.58 (0.85–2.92), *p* = 0.146	1.56 (0.84–2.90), *p* = 0.157	1.53 (0.82–2.84), *p* = 0.178	1.52 (0.82–2.83), *p* = 0.184	—
High vs. low NLR (≥3.15)	—	—	1.74 (0.97–3.11), *p* = 0.061	1.60 (0.87–2.93), *p* = 0.129	1.55 (0.83–2.89), *p* = 0.171	1.34 (0.75–2.41), *p* = 0.327
High vs. low albumin (≥3.85 g/dL)	—	—	—	0.71 (0.39–1.32), *p* = 0.279	0.71 (0.39–1.32), *p* = 0.281	0.66 (0.36–1.21), *p* = 0.179
High vs. low LDH (≥227 U/L)	—	—	—	—	1.13 (0.63–2.02), *p* = 0.685	—
Bone metastasis (present vs. absent)	—	—	—	—	—	1.27 (0.71–2.27), *p* = 0.423
−2 Log Likelihood	**384.841**	**382.615**	**379.002**	**377.808**	**377.643**	**378.438**
Harrell’s C-index (95% CI)	**0.664 (0.584–0.721)**	**0.695 (0.615–0.770)**	**0.699 (0.631–0.790)**	**0.712 (0.638–0.790)**	**0.720 (0.639–0.801)**	**0.690 (0.624–0.783)**

Incremental model performance						
Sequential comparison					LR χ²	p-value
Model 2 vs. Model 1					**2.225**	**0.136**
Model 3 vs. Model 2					**3.613**	**0.057**
Model 4 vs. Model 3					**1.194**	**0.275**
Model 5 vs. Model 4					**0.165**	**0.685**

Model definitions.

• Model 1: IMDC risk classification only.

• Model 2: Model 1 + metastatic burden.

• Model 3: Model 2 + neutrophil-to-lymphocyte ratio.

• Model 4: Model 3 + serum albumin.

• Model 5: Model 4 + lactate dehydrogenase.

• Alternative Model: IMDC + bone metastasis + neutrophil-to-lymphocyte ratio + serum albumin.

HR, hazard ratio; CI, confidence interval; IMDC, International Metastatic Renal Cell Carcinoma Database Consortium; NLR, neutrophil-to-lymphocyte ratio; LDH, lactate dehydrogenase.

Sequential multivariable Cox regression analyses were performed using a common complete-case cohort (n = 102; 55 deaths) to ensure direct comparability across all models. Model performance was evaluated using Harrell’s concordance index (C-index), −2 Log Likelihood (−2LL), and likelihood-ratio (LR) tests. Approximate 95% confidence intervals for Harrell’s C-index were estimated using 1,000 bootstrap resamples. Model 1 included IMDC risk classification only. Model 2 additionally incorporated metastatic burden. Model 3 further included neutrophil-to-lymphocyte ratio (NLR). Albumin and lactate dehydrogenase (LDH) were sequentially added in Models 4 and 5, respectively. The alternative model replaced metastatic burden with bone metastasis status while retaining the remaining variables. Higher C-index values indicate better model discrimination, whereas lower −2LL values indicate better model fit. Bold values indicate model-performance and model-comparison statistics.

The sequential addition of metastatic burden (Model 2), NLR (Model 3), albumin (Model 4), and LDH (Model 5) resulted in modest numerical improvements in model discrimination. Harrell’s C-index increased from 0.664 in Model 1 to 0.695, 0.699, 0.712, and 0.720 in Models 2 through 5, respectively. Model fit also improved numerically, as reflected by the progressive reduction in −2 Log Likelihood from 384.841 in Model 1 to 377.643 in Model 5. However, likelihood-ratio testing showed that none of the sequentially expanded models significantly improved model performance compared with the preceding model (Model 2 vs. Model 1, Δχ²=2.225, p=0.136; Model 3 vs. Model 2, Δχ²=3.613, p=0.057; Model 4 vs. Model 3, Δχ²=1.194, p=0.275; Model 5 vs. Model 4, Δχ²=0.165, p=0.685).

In the fully expanded Model 5, multiple-site metastatic disease (HR = 1.52, 95% CI: 0.82–2.83, p=0.184), elevated NLR (HR = 1.55, 95% CI: 0.83–2.89, p=0.171), higher albumin level (HR = 0.71, 95% CI: 0.39–1.32, p=0.281), and elevated LDH (HR = 1.13, 95% CI: 0.63–2.02, p=0.685) were not independently associated with overall survival after adjustment for IMDC risk classification.

In the alternative model, metastatic burden was replaced with bone metastasis. Bone metastasis was not independently associated with overall survival (HR = 1.27, 95% CI: 0.71–2.27, p=0.423). The alternative model yielded a −2 Log Likelihood of 378.438 and a Harrell’s C-index of 0.690, indicating lower discriminatory performance than the corresponding metastatic-burden model, while remaining numerically superior to the IMDC-only model.

## Discussion

In this real-world cohort of patients with metastatic renal cell carcinoma (mRCC), IMDC risk classification remained the strongest independent prognostic determinant of overall survival. Although sequential incorporation of metastatic burden and host-related biomarkers produced modest numerical improvements in model discrimination, these expanded variables did not demonstrate independent prognostic value beyond IMDC in multivariable analyses. These findings support the continued central role of the IMDC model for prognostic stratification while suggesting that the additional variables evaluated in this study provide only limited incremental prognostic information.

Our findings further reinforce the established prognostic role of the IMDC risk classification in patients with mRCC. Across all multivariable models, IMDC poor-risk classification consistently remained the strongest independent predictor of adverse survival outcomes, confirming its robustness despite the sequential incorporation of metastatic burden and host-related biomarkers. These findings are in agreement with the original IMDC model and subsequent validation studies, which demonstrated that IMDC remains the most widely accepted and clinically applicable prognostic model in the contemporary management of mRCC (6–9). Although the expanded models demonstrated modest numerical improvements in discriminatory performance, none of the additional variables provided statistically significant incremental prognostic value beyond IMDC after multivariable adjustment. This observation suggests that the IMDC model continues to capture most of the clinically relevant prognostic information in routine practice and remains an appropriate foundation for prognostic stratification in patients with mRCC.

Recent studies have increasingly investigated inflammatory and nutritional biomarkers as potential adjunctive prognostic variables in mRCC (9–13). Among these, NLR has emerged as one of the most extensively investigated biomarkers because it may capture interactions between tumor-associated inflammation and host immune response (10, 11). In the current study, elevated NLR was significantly associated with shorter OS in Kaplan–Meier and univariate Cox analyses. Moreover, incorporation of NLR into the sequential expanded models resulted in a modest numerical improvement in Harrell’s C-index. However, its prognostic effect lost statistical significance after adjustment for IMDC and metastatic burden in multivariable analyses. This finding suggests that although NLR may carry prognostic information, its additive prognostic utility beyond established IMDC-based stratification appears relatively restricted. Additionally, NLR may partially reflect components of systemic disease burden already captured within established IMDC risk classification. The use of cohort-specific median values is consistent with exploratory prognostic analyses in which universally accepted biomarker thresholds are lacking; however, externally validated cut-off values would be preferable for future clinical implementation.

Similarly, albumin and LDH did not demonstrate independent prognostic significance in expanded multivariable models despite modest numerical contributions to C-index values. Serum albumin has previously been associated with nutritional status, systemic inflammation, and cancer-related cachexia in mRCC (12), whereas elevated LDH has been linked to aggressive tumor biology and inferior oncologic outcomes (13). Although albumin demonstrated significant separation of Kaplan–Meier survival curves and was associated with improved overall survival in univariate analysis, this association was attenuated after multivariable adjustment. Nevertheless, our findings suggest that these biomarkers may provide only limited incremental prognostic value beyond IMDC in routine clinical practice. Importantly, the fully expanded model incorporating metastatic burden, NLR, albumin, and LDH achieved the highest C-index (0.720), indicating that combined incorporation of host-related variables may modestly improve survival discrimination numerically even in the absence of statistically independent prognostic significance.

Metastatic burden and metastatic pattern have emerged as additional variables potentially associated with disease aggressiveness in mRCC (14, 15). Previous studies have demonstrated that multiple metastatic involvement and bone metastasis may be associated with inferior survival outcomes (16, 17). In our analysis, patients with multiple metastatic sites and bone metastasis demonstrated numerically shorter overall survival; however, neither variable remained independently associated with survival in the expanded multivariable models. The absence of independent prognostic significance may partly reflect overlap with IMDC-associated clinical risk features. Interestingly, replacing metastatic burden with bone metastasis in the alternative model resulted in slightly lower discriminatory performance compared with the corresponding metastatic-burden model. The numerically higher discrimination observed in the metastatic-burden model may suggest that overall disease extent captures prognostic information more comprehensively than isolated bone involvement; however, this finding should be interpreted cautiously.

In the present study, sequential incorporation of metastatic burden and host-related biomarkers resulted in modest numerical improvements in model discrimination. However, these incremental gains did not reach statistical significance, and IMDC consistently remained the strongest independent prognostic determinant across all multivariable models. These findings are consistent with recent validation studies of the MEET-URO score, which demonstrated that incorporation of pretreatment neutrophil-to-lymphocyte ratio and bone metastasis status into the IMDC classification provides modest improvements in prognostic discrimination across different first-line immunotherapy settings while preserving IMDC as the principal prognostic framework (18, 19). Collectively, these findings suggest that readily available host-related biomarkers may provide complementary prognostic information, although their incremental contribution beyond IMDC appears to be limited.

One of the major strengths of the present study is the sequential model-based approach evaluating the incremental contribution of expanded variables beyond IMDC using Harrell’s C-index, likelihood-ratio testing, and model fit statistics. Rather than focusing solely on the statistical significance of individual variables, our analysis specifically evaluated whether the sequential incorporation of additional variables improved overall model discrimination and prognostic performance. This sequential modeling strategy may provide a more clinically relevant assessment of incremental prognostic utility beyond conventional biomarker analyses in real-world clinical practice.

The present findings should be interpreted in light of several methodological limitations. First, the retrospective single-center design may have introduced selection bias and may limit the generalizability of our findings. Second, the relatively modest sample size and limited number of outcome events may have reduced statistical power and increased the risk of model overfitting, particularly in multivariable analyses. Therefore, the observed model performance should be considered exploratory rather than definitive. Third, treatment heterogeneity over the study period may have influenced survival outcomes. Finally, molecular and genomic biomarkers, which may further refine prognostic stratification, were not available for analysis.

Despite these limitations, our findings provide clinically relevant real-world evidence regarding the prognostic performance of expanded models in metastatic RCC. Recent real-world validation studies have suggested that inflammatory biomarkers and metastatic burden may provide incremental prognostic information beyond the IMDC classification in patients receiving contemporary systemic therapies (20, 21). Although these studies reported incremental prognostic value, the magnitude of improvement remained relatively modest, which is consistent with the findings of the present study. Consistent with these observations, our results demonstrated modest numerical improvements in prognostic discrimination after sequential incorporation of metastatic burden and readily available laboratory biomarkers. However, these incremental improvements did not reach statistical significance, while IMDC remained the dominant prognostic framework in routine clinical practice. Nevertheless, external validation in larger, preferably multicenter cohorts is required before these expanded prognostic models can be considered for broader clinical application. Future studies integrating molecular, genomic, and clinical biomarkers may further clarify whether expanded prognostic models can meaningfully improve survival discrimination beyond IMDC.

## Conclusion

In this real-world cohort of patients with metastatic renal cell carcinoma, IMDC risk classification remained the most consistent and clinically robust determinant of overall survival. Although expanded prognostic models incorporating metastatic burden, inflammatory, and nutritional biomarkers demonstrated modest numerical improvements in survival discrimination, these variables did not provide statistically significant incremental prognostic value beyond IMDC. The findings of the present study support the continued central role of IMDC in routine clinical practice while suggesting that expanded prognostic models may offer only limited incremental prognostic benefit in real-world mRCC populations. Further multicenter studies integrating molecular and clinical biomarkers are warranted to refine prognostic stratification beyond conventional IMDC-based models.

## Data Availability

The original contributions presented in the study are included in the article/supplementary material. Further inquiries can be directed to the corresponding author.
